# Supplementary material for: Categorical identity signatures can reduce host error rates during brood parasitism
Source: PLoS Biol. 2026 Feb 20;24(2):e3003667. doi: 10.1371/journal.pbio.3003667 (PMC12952647; doi:10.1371/journal.pbio.3003667)
Supplement: S1 Text — Fig A. Histograms illustrating distributions of traits in the cuckoo finch gens parasitizing zitting cisticolas. The three traits illustrated are those that predict rejection in zitting cisticolas: color, marking luminance, and mean feature size. Insets show representative egg phenotypes from near the ends of each distribution. The data underlying this figure are available at https://doi.org/10.17863/CAM.116928.2. Fig B. Pattern differences between cuckoo finch (n = 129) and prinia (n = 371) eggs in the present dataset. Differences were similar to those found in reference [44] on a dataset of prinia and cuckoo finch eggs measured at the same study site in 2007–2009. P-values refer to Wilcoxon rank-sum tests. Whiskers extend to the most extreme value within 1.5 * IQR (inter-quartile range) of the corresponding hinge of the box. Hinges correspond to first and third quartiles; the IQR is the distance between the first and third quartiles. The data underlying this figure are available at https://doi.org/10.17863/CAM.116928.2. Fig C. The summary of simulated results with various shapes of categorical distributions. (a–e) The genotype distributions, where increasing distance between the two categorical peaks results in decreased width of each peak (such that the overall host genotypic variation is constant). From left to right, the categorical distribution becomes more discrete, resulting in greater differences in (f–j) success rate, (k–o) Type I error rate, and (p–t) Type II error rate between the respective categorical and continuous populations. In many instances, the categorical population exhibits greater success than the continuous population. Note that panels d, i, n, and s illustrate the same results as shown in Fig 4a-c, and are included here for completeness. The simulated data underlying this figure are available at https://doi.org/10.17863/CAM.116928.2. Table A. Frequencies of clutches of each category in zitting cisticolas, according to the human eye and PAM [file pbio.3003667.s001.docx]

**Supplementary Information**

1. **Quantifying egg phenotypes**
2. **Colour**

Background colour of eggs was measured as in reference [1]; briefly, reflectance spectra were obtained using an Ocean Optics USB4000 Spectrophotometer, with a PX-2 pulsed xenon light source and an R400-7-UV/VIS reflectance probe, standardised with a Spectralon 99% White Standard (Labsphere) and a black felt cloth. The average of five measurements of an egg’s background colour was taken and photon catches were calculated using the R package PAVO [2].

We calculated chromatic and achromatic contrasts (measured in just noticeable differences; JNDs) between different eggs using the receptor noise limited model, which simulates early-stage visual processing [3]. JND values above one are considered discriminable under ideal viewing conditions, with higher values correlating with higher discriminability. A Weber fraction of 0.05 [4], and photoreceptor densities for the blue tit model [5] were used.

1. **Pattern**

RAW (linearised) images of eggs were taken, and normalised, scaled, and converted to greyscale exactly as described in [6–8]. We quantified pattern traits using the same methods as reference [7].

In brief, we used granularity analysis [1,9–11] in MATLAB to quantify measures of principal marking size, variation in marking size, and total pattern contrast. In reference [1], this granularity analysis was conducted on three 5 mm x 5 mm squares at the wide, middle, and narrow regions of each egg. Here, we used only two 5 mm x 5 mm squares (at the wide and narrow regions), because many eggs were too small for three non-overlapping regions to be selected [7].

We used the adaptive thresholding tool [12] in the MICA toolbox [13] to quantify total pattern coverage on the egg’s surface, and the dispersion of pattern (the extent to which pattern is dispersed between narrow and wide poles of the egg). Thresholding parameters were chosen such that, to the human eye, regions of pattern were most accurately defined: High Pass = 50, Low Pass = 1, Threshold = 0.9. Pattern coverage was defined as the area of the thresholded pattern region divided by the area of the egg. Pattern dispersion was defined as the pattern coverage at the narrow pole subtracted from the pattern coverage at the blunt pole [7]. This again differs from the method used in reference [1], in which a different thresholding method was used, and pattern coverage was measured for the three square regions rather than the whole egg.

We used the scale-invariant feature transform (SIFT) algorithm in NaturePatternMatch [14] (NPM) to identify ‘SIFT features’, which correspond loosely to individual pattern markings. We quantified the number of SIFT features as in reference [7] and the mean of the size of the SIFT features as in reference [6]. Features larger than 25 and smaller than 2 were excluded from feature size quantification, since these correspond to the entire egg or to noise.

One measure not extracted in previous studies was a measure of marking luminance, independent of background egg colour. To measure marking luminance, we selected three regions of each egg by using a random number generator to specify coordinates on the egg surface. The luminance value of the pattern marking nearest to each region was measured, with the mean of these three values taken to produce a measure of luminance of the egg pattern. Luminance values can be extracted using the red, green, or blue channels of RGB images. We arbitrarily used the green channel, though we confirmed that for each species, the pattern luminance values for the green channel were highly correlated (according to Pearson’s correlation tests; or Spearman’s correlation when pattern luminance values were not normally distributed) with values for the red and blue channels (all r > 0.90; all P < 0.001). We also used a UV camera as described for prinias in reference [7] to confirm that marking luminance values in UV channels were also correlated with those in visible channels for zitting cisticola eggs (again all r > 0.90; all P < 0.001).

1. **Details of the rejection models for croaking cisticolas**

As discussed in the methods section, we used two different models to test the traits predicting rejection in croaking cisticolas. This is because some croaking cisticola eggs were immaculate, and some traits cannot be calculated for immaculate eggs.

From 38 experiments conducted on croaking cisticolas in 2019, 2020, 2022, and 2023 in which no eggs were immaculate, the best model of rejection contained differences in colour and marking luminance as predictors (Colour: Estimate = 1.95 ±0.67SE, Z_35_ = 2.90, P = 0.003; Marking luminance: Estimate = 0.38 ±0.16SE, Z_35_ = 2.38, P = 0.02; R^2^ = 0.76).

When including the 12 other experiments in which either or both of the experimental egg and host eggs were immaculate, and therefore marking luminance could not be quantified, the best model of rejection contained differences in colour and pattern coverage as predictors (Colour: Estimate = 0.79 ±0.25SE, Z_47_ = 3.18, P = 0.002; Pattern coverage: Estimate = 30.83 ±14.46SE, Z_47_ = 2.13, P = 0.03; R^2^ = 0.43).

1. **Cluster analysis on egg colour**

For pattern, PAM (Partitioning Around Medioids) cluster analyses were performed on each pattern trait. However, this could not be done for colour because background colour was measured as predicted relative cone catch values for the four avian cone types (UVS, SWS, MWS, and LWS cones). Therefore, the first or first and second principal components of these four variables was used as a variable corresponding to colour. Principal components (or analogous dimensionality-reduction measures) can reduce dimensionality and account for collinearity in cone catch values, and have therefore been used to quantify colour according to organisms’ visual systems in studies on a range of taxa [15–19]. Using one or two principal components of cone catch values allows us to study the main axis or axes of colour variation in systems. The choice of one or two principal components in the present study depended on the percentage of variation predicted by either one or two principal components, since we aimed to conduct cluster analyses on the main axes of variation in colour space.

For zitting cisticolas, a principal component analysis on egg colour showed that the first principal component explained 74.3% of the variance. This principal component was used in the cluster analysis, and corresponded well to the main axis of variation observed in zitting cisticola eggs: namely, blue to white (Figs 1a and 2a).

For croaking cisticolas, a principal component analysis on egg colour (measured as the photon catch values for UVS, SWS, MWS, and LWS cones) showed that the first principal component explained 81.6% of the variance. This principal component was used in the cluster analysis, and corresponded well to the main axis of variation observed in croaking cisticola eggs: namely, blue to white (Fig 2b).

For prinias, a principal component analysis on egg colour showed that the first principal component explained 60.1% of the variance, and the first and second principal components together explained 98.0% of the variation. The first two principal components were used in the cluster analysis, and corresponded well to the main axes of variation in prinias as described in references [1,20,21]: namely red or olive to blue or white (Figs 1b and 2c).

For red-faced cisticolas, egg colour did not predict rejection so no cluster analysis nor principal component analysis was done on this trait.

Results of cluster analyses are provided in the main text. In addition to the results reported in main text, we tested for clustering in the three traits in a dataset on prinias published in references [6,8], from an earlier time period for which we lacked comparable rejection data (2018–2020). In this dataset, the results were qualitatively similar to those reported for 2022–2023: for each trait there was no evidence that the number of clusters differed from one (colour: P = 0.23; pattern coverage: P = 0.09; pattern dispersion: P = 0.63).

We also counted the total number of zitting cisticola clutches of each category. Eggs were broadly equally distributed across the six categories, though not precisely: white eggs with pale blotches were just under twice as frequent as each of the other categories. The frequencies of each category are given in Table A. The clustering algorithm assigned one egg as having dark blotches, while to the human eye all eggs with dark markings had small speckles, illustrating that although the clustering algorithm corresponded well with human classification, they were not identical (Fig 2a).

**Table A.** Frequencies of clutches of each category in zitting cisticolas, according to the human eye and PAM clustering.

| **Category** | **Frequency according to human eye** | **Frequency according to clustering algorithm** |
| --- | --- | --- |
| White, dark speckles | 15 | 16 |
| Blue, dark speckles | 15 | 16 |
| White, pale speckles | 18 | 21 |
| Blue, pale speckles | 22 | 17 |
| White, pale blotches | 32 | 32 |
| Blue, pale blotches | 17 | 16 |
| White, dark blotches | 0 | 1 |

1. **Direct tests for multimodality support conclusions of cluster analysis**

Alongside cluster analyses, we used Hartigans’ dip tests and Silverman’s tests to test for multimodality (see Methods).

For zitting cisticolas, cluster analyses suggested that the three traits predicting rejection were best described by two clusters each. For all three traits, Hartigans’ dip tests all rejected the null hypothesis of unimodality (colour: D = 0.05, P = 0.04; marking luminance: D = 0.05, P < 0.001; marking size: D = 0.03, P = 0.03). Similarly, Silverman’s tests indicated that each distribution had more than one mode (colour: Critical bandwidth = 0.99, P < 0.001; marking luminance: Critical bandwidth = 4.09, P < 0.001; marking size: Critical bandwidth = 0.29, P < 0.001), but not more than two modes (colour: Critical bandwidth = 0.32, P = 0.74; marking luminance: Critical bandwidth = 0.85, P = 0.88; marking size: Critical bandwidth = 0.10, P = 0.70). Thus, the results of tests for multimodality fully conformed with the results from cluster analyses.

For croaking cisticolas, cluster analyses suggested that, of the three traits predicting rejection, colour was best described by two clusters each, while pattern coverage and marking luminance were best described by one cluster. This was somewhat supported by tests for multimodality. For colour, although Hartigans’ dip test did not reject the null hypothesis of unimodality (D = 0.047; P = 0.37), Silverman’s tests suggested that the number of modes marginally significantly differed from one (critical bandwidth = 0.88; P = 0.05), but not two (critical bandwidth = 0.42; P = 0.65). Thus, there was some evidence that colour in croaking cisticolas is multimodally distributed. For pattern coverage, both Hartigans’ dip tests (D = 0.061; P < 0.001) and Silverman’s tests (Critical bandwidth = 0.015; P < 0.001) indicated that there was more than one mode, though not more than two modes (Critical bandwidth = 0.0080; P = 0.97). This result differed from cluster analysis. Using the *locmodes* function in the *multimode* package, the two located modes can be visualized – these coincided with the two peaks in the histogram in Fig 2b. In other words, tests for multimodality indicated that the relatively few immaculate eggs in the population constituted a separate mode, while cluster analysis did not assign immaculate eggs to a separate cluster to patterned eggs. For pattern luminance, neither Hartigans’ dip test (D = 0.028; P = 0.48) nor Silverman’s test rejected the null hypothesis of unimodality (Critical bandwidth = 3.06; P = 0.07). Therefore, there were some inconsistencies between the results of cluster analysis and tests for multimodality (and even between the two tests for multimodality themselves). However, together the tests indicated that croaking cisticolas exhibited a mixture of continuous and categorical traits, disagreeing only on which traits were continuous and which were categorical.

For prinias, cluster analyses suggested that the traits predicting rejection (pattern coverage, pattern dispersion, and colour) were continuously distributed. For pattern coverage, both Hartigans’ dip test (D = 0.015; P = 0.70) and Silverman’s test (Critical bandwidth = 0.015; P = 0.34) did not reject the null hypothesis of unimodality. For pattern dispersion, again both Hartigans’ dip test (D = 0.016; P = 0.54) and Silverman’s test (Critical bandwidth = 0.020; P = 0.40) did not reject the null hypothesis of unimodality. For colour, one can only conduct tests of multimodality on each principal component separately (which limits the comparability of the cluster analysis and the tests of multimodality). Nevertheless, for PC1, both Hartigans’ dip test (D=0.015; P=0.98) and Silverman’s test (Critical bandwidth = 0.38; P = 0.38) did not reject the null hypothesis of unimodality. For PC2, again both Hartigans’ dip test (D = 0.020; P = 0.73) and Silverman’s test (Critical bandwidth = 0.34; P = 0.25) did not reject the null hypothesis of unimodality. Thus, the results of tests for multimodality fully conformed with the results from cluster analyses.

For red-faced cisticolas, cluster analyses suggested that the traits predicting rejection (marking luminance and number of SIFT features) were continuously distributed. For pattern coverage, both Hartigans’ dip test (D = 0.022; P = 0.83) and Silverman’s test (Critical bandwidth = 0.89; P = 0.17) did not reject the null hypothesis of unimodality. For the number of SIFT features, again both Hartigans’ dip test (D = 0.018; P = 0.98) and Silverman’s test (Critical bandwidth = 11.8; P = 0.64) did not reject the null hypothesis of unimodality. Thus, the results of tests for multimodality fully conformed with the results from cluster analyses.

Overall, barring some discrepancies for some of the traits of croaking cisticolas, results from tests for multimodality fully conformed with those of cluster analysis, indicating the robustness of each classification measure (see Table B for easy comparison).

**Table B.** Comparison of results from cluster analyses and direct tests for multimodality. Conclusions from each test are provided alongside P-values (see main text and S1 Text §d for details including test statistics and sample sizes).

| **Species** | **Trait** | **Cluster analysis conclusion**  **(P-value)** | **Hartigans’ dip test conclusion**  **(P-value)** | **Silverman’s test conclusion**  **(P-value)** |
| --- | --- | --- | --- | --- |
| Zitting cisticola | Colour | Categorical (<0.01) | Categorical (0.04) | Categorical (<0.001) |
|  | Marking luminance | Categorical (<0.01) | Categorical (<0.001) | Categorical (<0.001) |
|  | Marking size | Categorical (<0.01) | Categorical (0.03) | Categorical (<0.001) |
|  |  |  |  |  |
| Croaking cisticola | Colour | Categorical (0.03) | Continuous (0.37) | Categorical^*^ (0.05) |
|  | Pattern coverage | Continuous (0.51) | Categorical (<0.001) | Categorical (<0.001) |
|  | Marking luminance | Continuous (0.16) | Continuous (0.48) | Continuous (0.07) |
|  |  |  |  |  |
| Prinia | Colour | Continuous (0.06) | Continuous^†^ (0.98) | Continuous^†^ (0.38) |
|  | Pattern coverage | Continuous (0.18) | Continuous (0.70) | Continuous (0.34) |
|  | Pattern dispersion | Continuous (0.89) | Continuous (0.54) | Continuous (0.40) |
|  |  |  |  |  |
| Red-faced cisticola | Marking luminance | Continuous (0.44) | Continuous (0.83) | Continuous (0.17) |
|  | No. SIFT features | Continuous (0.09) | Continuous (0.98) | Continuous (0.64) |
|  |  |  |  |  |

^*^ P-value marginally significant.

^†^ Only results for first principal component of colour provided (see S1 Text §d for details).

1. **Why we might expect a trade-off between Type I and Type II errors**

As discussed in the main text, previous work has shown that a trade-off between Type I and Type II errors should be expected because discrimination thresholds have opposing effects on the two types of error [22–24]. Hosts with lower thresholds (i.e., hosts that are highly discriminating) will make more Type I errors but fewer Type II errors. This is because such hosts will be able to discriminate small differences between eggs, resulting in them often rejecting even closely mimetic parasitic eggs (and thus making few Type II errors). However, if one of such a host’s own eggs is slightly aberrant, then it is also likely to fall outside the low rejection threshold, and therefore be rejected. Thus, hosts with low rejection thresholds should make more Type I errors than hosts with higher rejection thresholds. Similarly, hosts with higher thresholds (i.e. hosts that are poor at discriminating small differences) should make fewer Type I errors, since even aberrant eggs within their own clutches are unlikely to be rejected. However, they should make more Type II errors, since even poorly-mimetic parasitic eggs will often fall within the large rejection threshold. This argument applies both for strict thresholds (i.e. where hosts always accept eggs that fall within thresholds and always reject eggs that do not), and for less strict decision-making criteria (for instance where probability of rejection increases with increased difference between eggs, but without a sharp cut-off where all eggs either side of the cut-off are rejected or accepted).

More recently, a second cause of the trade-off between Type I and Type II errors has been established. This cause is the trade-off between phenotypic consistency within clutches and phenotypic distinctiveness between clutches, which likely arises for mechanistic reasons: mechanisms that generate repeatability and therefore consistency within individuals are likely to result in lower distinctiveness between individuals [6,25]. The consistency–distinctiveness trade-off has been shown between species in the Ploceidae and Cisticolidae, and within one species: the tawny-flanked prinia [6,25]. Importantly, this trade-off means that our finding that zitting cisticolas show a surprisingly low rate of *both* Type I and Type II errors cannot simply be a result of them having both lower intra-individual variation (i.e., higher consistency) and higher inter-individual distinctiveness than other hosts. Here, we provide further demonstration of this trade-off between consistency and distinctiveness in the two most common hosts we studied: zitting cisticolas and tawny-flanked prinias. We conducted this analysis on egg background colour because this trait predicts rejection in both species.

One measure of egg phenotype distinctiveness is the amount of phenotypic space occupied by the eggs of a host species. To the human eye, zitting cisticolas seem to occupy less phenotypic space than tawny-flanked prinias. For instance, zitting cisticola eggs are either blue or white, whereas prinia eggs encompass these colours along with red and olive [1] (Fig 1). To test whether prinias occupied greater phenotypic space in background colour, we used the *voloverlap* function in the R package PAVO [2] to quantify the volume of overlap in avian colour space occupied by these two species. We had n = 119 colour measurements for zitting cisticola eggs, and n = 626 measurements for prinia eggs; therefore, we calculated the volume occupied by a randomly selected subset of n = 119 prinia eggs to ensure it was comparable to the dataset of zitting cisticola egg colours. Prinias occupied more phenotypic space than did zitting cisticolas (1.91*10^-4^ and 3.29*10^-5^ respectively). In other words, prinia eggs exhibited higher distinctiveness in colour than did zitting cisticola eggs.

By contrast, zitting cisticolas showed greater consistency in egg colour than did prinias (mean intra-clutch JNDs = 0.620 and 1.82 respectively). Therefore, in this trait that can be compared between the two species, prinias showed both lower consistency and higher distinctiveness than did zitting cisticolas, further illustrating the trade-off between consistency and distinctiveness described and tested more broadly in references [6,25]. This trade-off indicates that the low rates of Type I and Type II errors in zitting cisticolas cannot simply be attributed to the extent of variation within and between clutches in this species.

1. **Phenotypes of cuckoo finch eggs laid in zitting cisticola eggs**

As discussed in the main text, cuckoo finch females specialise on a particular host species, leading to ‘gentes’ (singular gens; defined as a maternal lineage that targets a particular host) [21]. Eggs of cuckoo finches parasitising zitting cisticolas are less well described than those of other cuckoo finch gentes [1,7,15,21], so we provide a more thorough description here. Cuckoo finch eggs laid in zitting cisticola nests vary in the same traits used by zitting cisticolas in rejection (Fig A), namely background colour (white to blue), marking luminance (very dark brown to pale brown, though there were very few eggs with dark markings in the present sample), and marking size (small speckles to large blotches). The diversity of cuckoo finch phenotypes laid in zitting cisticola nests in 2018–2023 was greater than that in 2012–2014, when only white eggs with pale brown speckles were encountered [21].


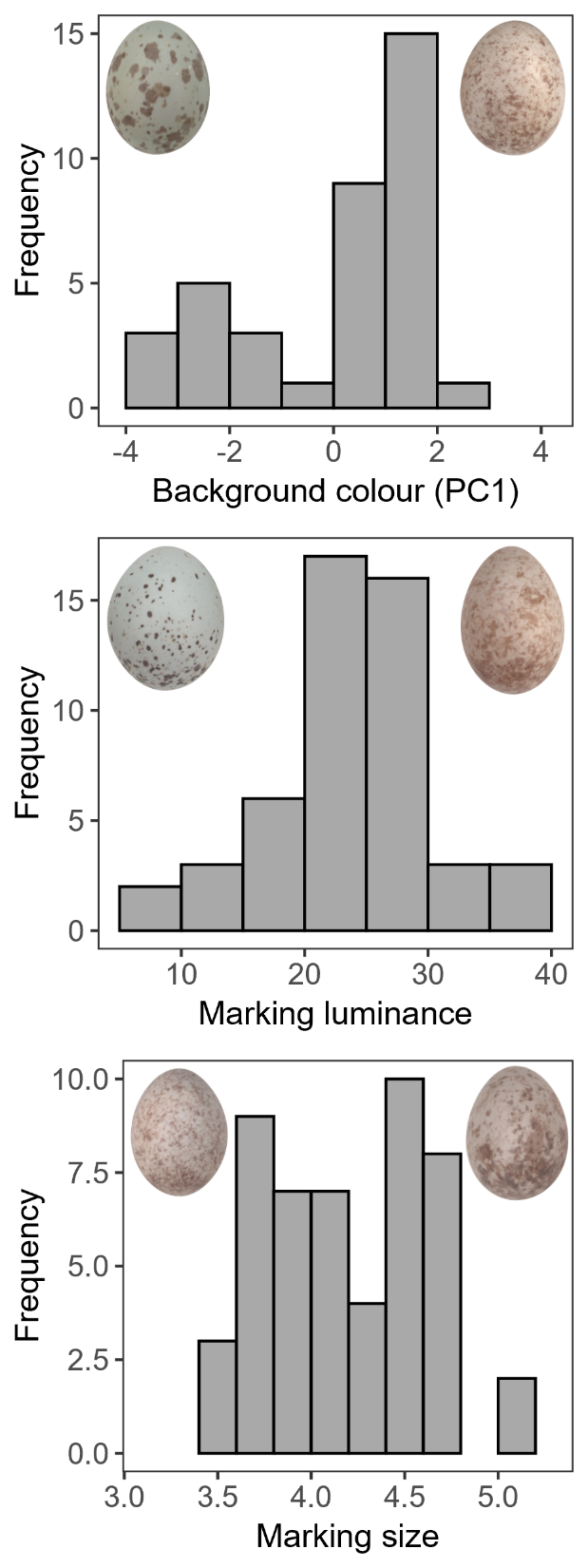


**Fig A.** Histograms illustrating distributions of traits in the cuckoo finch gens parasitising zitting cisticolas. The three traits illustrated are those that predict rejection in zitting cisticolas: colour, marking luminance, and mean feature size. Insets show representative egg phenotypes from near the ends of each distribution. The data underlying this figure are available at <https://doi.org/10.17863/CAM.116928.2>.

1. **Detailed discussion of error rates and comparisons with previous work**

Several results from this study diverge partly from previous studies on this system. For example, the traits which predicted rejection in prinias and red-faced cisticolas in this dataset were not identical to those that have previously been shown to predict rejection in these species at the same study site in 2007–2009 [1,15]. Furthermore, while these previous studies estimated Type II error rates of approximately 50% in both prinias and red-faced cisticolas, here, parametrising a rejection model with real phenotypes estimated a Type II error rate of approximately 34% in prinias. This suggests that, in the intervening time since egg rejection experiments were conducted in 2007–2009 [1,15], there may have been a change in which traits hosts use in rejection decisions and their rejection accuracy. While only correlational, we may speculate that this is (in part) due to variation in parasitism rates. For example, high parasitism rates in the early 2010s [7,21] could have driven hosts to become more discriminating, resulting in fewer Type II errors and changes in the traits used in decision-making. Perhaps changes in cuckoo finch egg phenotypes and the frequency of different cuckoo finch phenotypes (some of which have already been documented [20,26]), or sampling effects (i.e., which individual hosts and parasites happened to be present in the study area each year) could additionally explain differences in experimental results between this study and previous studies. Alternatively (and not mutually exclusively), this study involved slightly different methods to those used in previous studies. For example, measures from NPM such as the number of SIFT features were extracted in this study but not in previous studies, and these proved to be informative for red-faced cisticolas. Similarly, the thresholding method used to extract measures of pattern coverage and pattern dispersion differed slightly between this study and previous studies (see S1 Text §a); these measures proved to be informative for prinias. In general, such discrepancies illustrate the importance of replicating previous studies, since behaviours, traits, or methods for measuring traits may have changed in the intervening time.

Despite the differences we found in which traits predicted rejection, cuckoo finches differed on average from prinias in exactly the same traits as previously shown [1]. Fig B shows comparisons of pattern traits in cuckoo finches and prinias, and illustrates that cuckoo finches differed significantly from prinias in pattern dispersion, variation in marking size (termed ‘proportion energy’ in reference [1]), and principal marking size. These are the same three traits (measured using the same or slightly different methods; see S1 Text §a) previously found to differ between the species, and also the three pattern traits that predicted rejection in this earlier dataset [1]. Meanwhile, the trait that predicted rejection in the present dataset but not in reference [1] – pattern coverage – differed between prinias and cuckoo finches in neither dataset (Fig B). It is also surprising that although prinias still differ from cuckoo finches in principal marking size and variation in marking size, these traits now do not predict rejection in the system, suggesting that imperfect mimicry provides prinias with information that they are not currently using (c.f. [7]).

One host species, the croaking cisticola, showed a mix of categorical and continuous variation in different traits predicting rejection behaviour. Accordingly, it exhibited similar estimated and observed Type II error rates to the continuously varying species (prinias and red-faced cisticolas), and similar estimated Type I rejection rates to the categorically varying species (the zitting cisticola). However, the observed Type I error rate in croaking cisticolas was higher than other species (Fig 3), and three out of six Type I errors occurred when the croaking cisticola also rejected the experimental egg (a higher proportion than for other hosts: respectively 4/15, 0/1, and 0/3 for prinias, zitting cisticolas, and red-faced cisticolas). Perhaps croaking cisticolas, having large bills compared to most other Cisticolidae [27], may damage (and therefore subsequently remove) their own eggs more often when turning them, or when rejecting a strong-shelled cuckoo finch egg [28] (as we have observed anecdotally), leading to an unexpectedly high number of observed Type I errors relative to other species. This suggests that morphological as well as perceptual constraints on error rates need to be considered when studying discrimination between self and non-self.


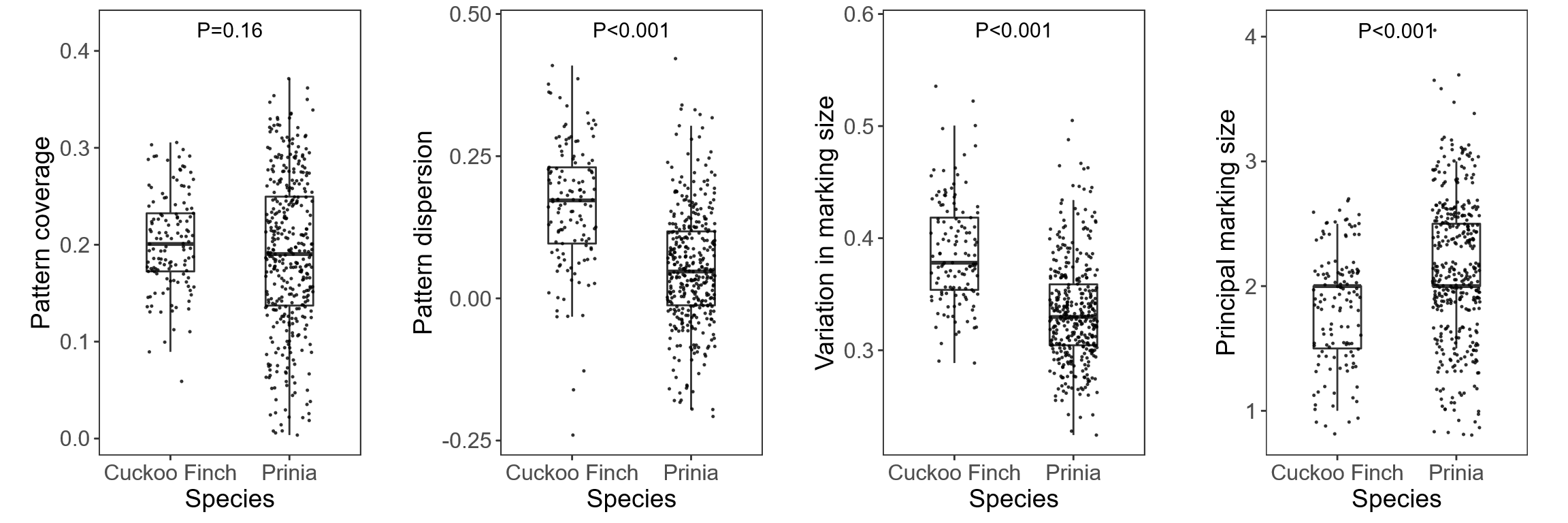


**Fig B.** Pattern differences between cuckoo finch (n = 129) and prinia (n = 371) eggs in the present dataset. Differences were similar to those found in reference [1] on a dataset of prinia and cuckoo finch eggs measured at the same study site in 2007–2009. P-values refer to Wilcoxon rank-sum tests. Whiskers extend to the most extreme value within 1.5 * IQR (inter-quartile range) of the corresponding hinge of the box. Hinges correspond to first and third quartiles; the IQR is the distance between the first and third quartiles. The data underlying this figure are available at <https://doi.org/10.17863/CAM.116928.2>.

1. **Details of individual-based simulations**
2. **Parameters for individual-based simulations**

Table C lists the parameters involved in the simulations and their chosen values (see Methods). Some of these values were varied in order to test the generalisability of results (see section ii).

**Table C.** Values and descriptions of parameters in individual-based simulations.

| Parameter name | Value | Description |
| --- | --- | --- |
| $N_{nest}$ | 10^3^ | Number of nests in the population |
| $N_{rep}$ | 10^3^ | Number of replicates in each simulation |
| $N_{egg, host}$ | 3 | Number of host eggs laid by each host individual |
| $N_{egg, para}$ | 1 | Number of parasite eggs laid when the nest is parasitised |
| $p_{parasitised}$ | 0.5 | Probability of a nest getting parasitised |
| $\sigma_{cont}$ | 0.25 | Standard deviation of egg genotypes under the continuous setting, where genotypes follow a unimodal normal distribution |
| $d_{cat}$ | 0.18–0.25^*^ | Trait distance between the two categories in the categorical setting |
| $\sigma_{cat}$ | 0–0.1735^*^ | Standard deviation of egg genotypes under the categorical setting, where genotypes follow a mixture distribution of two normal distributions with equal variance |
| $\sigma_{host}$ | 0 or 0.25^†^ | Phenotypic variation within host clutches |

^*^ These variables modify the shape of categorical distributions, and the values are changed for each simulation.

^†^ For the simulation of category-based rejection in hosts exhibiting categorical variation (Fig 4d–f in main text), this parameter is set to 0, to simulate hosts recognising eggs as belonging to one category or another, and thus ignoring within-clutch variation.

1. **Results of the threshold-based rejection model**

All simulation results are summarised in Fig C. Because the difference between the categorical and continuous distributions is more dramatic on the right-hand side, we will begin from the right-most column (panels e, j, o, and t).

The most extreme case is seen when the categorical population is genetically binary (i.e., there are only two distinct genotypes present in the population; Fig Ce; $d_{cat}$= 0.25). This population has lower success (i.e., probability of host egg survival having accounted for both Type II and Type I errors) than the continuous population when the threshold distance is less than 0.25, which is the half distance between the two categories (Fig Cj). The categorical distribution results in greater success when the threshold is between 0.25 and 0.5. When the threshold distance is larger than 0.5, the continuous distribution again results in greater success, until the threshold distance is very large, when both strategies result in equally poor outcomes (Fig Cj). These differences are observed because the Type II curve for the categorical population is a step function due to the threshold encompassing either one or two of the peaks of the distribution (Fig Ct; also see Main Text).

Moving towards the left in Fig C, we gradually see a smoothening trend in the categorical case in all three rates (i.e., success, Type I error, and Type II error rates all become more similar to those for the continuous population; Fig C). This is because we are gradually increasing the genotypic variation within the categorical population, while simultaneously making the two categories more similar to each other. As a result, the differences observed between the categorical and the continuous populations become less pronounced as genotypic variation in each category increases (Fig Cf–j).

Across all simulation results shown in Fig C, the Type I error frequency is effectively unaffected by distribution shape. This is because the likelihood of a Type I error depends only on within-clutch variation and the rejection threshold, and not on the genotypic variation within the population. The within-clutch variation is kept constant and therefore the Type I error rate at a given threshold should not differ between categorical and continuous populations. Thus, the Type I error curves for categorical and continuous populations are effectively identical (Fig Ck–o; also Fig Dc) – though they are not precisely identical. This is because the simulation does not consider Type I errors if the parasitic egg is accepted (since all host eggs will fail to survive in such instances). In other words, more Type II errors will result in fewer Type I errors in the simulations, simply because the simulations exclude the opportunity for hosts that make a Type II error to compare their own eggs.

Overall, the frequency of Type II errors varies with the shape of trait distributions, even though the overall standard deviation is the same across all cases (Fig C). This means that, if within-clutch variation were to be lower in a categorical population than in a continuous population, or if rejection thresholds evolved to be more permissive due to lower Type II error rates, such categorical distributions could generate both fewer Type I and Type II errors than comparable continuous distributions. Thus, the expected trade-off between Type I and Type II errors could be overcome by trait distributions.

**
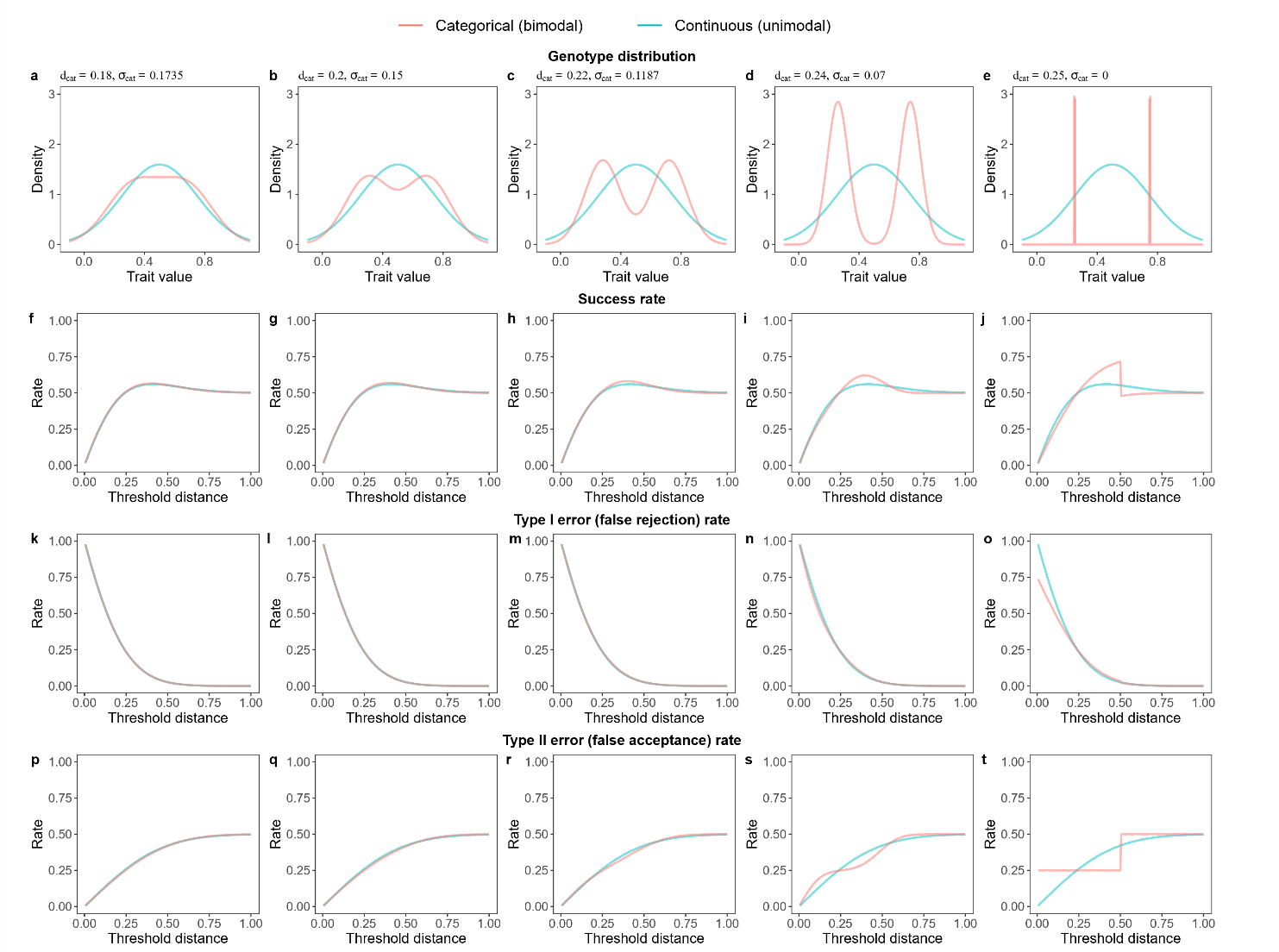
Fig C.** The summary of simulated results with various shapes of categorical distributions. (a–e) The genotype distributions, where increasing distance between the two categorical peaks results in decreased width of each peak (such that the overall host genotypic variation is constant). From left to right, the categorical distribution becomes more discrete, resulting in greater differences in (f–j) success rate, (k–o) Type I error rate, and (p–t) Type II error rate between the respective categorical and continuous populations. In many instances, the categorical population exhibits greater success than the continuous population. Note that panels d, i, n, and s illustrate the same results as shown in Fig a–c, and are included here for completeness. The simulated data underlying this figure are available at <https://doi.org/10.17863/CAM.116928.2>.

**Supplementary References**

1. Spottiswoode CN, Stevens M. Visual modeling shows that avian host parents use multiple visual cues in rejecting parasitic eggs. PNAS. 2010;107: 8672–8676. doi:10.1073/pnas.0910486107

2. Maia R, Gruson H, Endler JA, White TE. PAVO 2: New tools for the spectral and spatial analysis of colour in R. Methods Ecol Evol. 2019;10: 1097–1107. doi:10.1111/2041-210X.13174

3. Vorobyev M, Osorio D. Receptor noise as a determinant of colour thresholds. Proc R Soc Lond B Biol Sci. 1998;265: 351–358. doi:10.1098/rspb.1998.0302

4. Stoddard MC, Stevens M. Avian vision and the evolution of egg color mimicry in the common cuckoo. Evolution. 2011;65: 2004–2013. doi:10.1111/j.1558-5646.2011.01262.x

5. Hart NS, Partridge JC, Cuthill IC, Bennett ATD. Visual pigments, oil droplets, ocular media and cone photoreceptor distribution in two species of passerine bird: the blue tit (*Parus caeruleus* L.) and the blackbird (*Turdus merula* L.). J Comp Physiol A. 2000;186: 375–387. doi:10.1007/s003590050437

6. Dixit T, Chen K-C, Stoddard MC, Mahadevan L, Town CP, Spottiswoode CN. Repeatable randomness, invariant properties, and the design of biological signatures of identity. Evolution. 2023;77: 2224–2233. doi:10.1093/evolut/qpad134

7. Dixit T, Choi GPT, Al-Mosleh S, Lund J, Troscianko J, Moya C, et al. Combined measures of mimetic fidelity explain imperfect mimicry in a brood parasite-host system. Biol Lett. 2023;19: 20220538. Available: http://doi.org/10.1098/rsbl.2022.0538

8. Dixit T, Apostol AL, Chen K-C, Fulford AJC, Town CP, Spottiswoode CN. Visual complexity of egg patterns predicts egg rejection according to Weber’s law. Proc Biol Sci. 2022;289: 20220710. doi:10.1098/rspb.2022.0710

9. Barbosa A, Mäthger LM, Buresch KC, Kelly J, Chubb C, Chiao C-C, et al. Cuttlefish camouflage: The effects of substrate contrast and size in evoking uniform, mottle or disruptive body patterns. Vision Res. 2008;48: 1242–1253. doi:https://doi.org/10.1016/j.visres.2008.02.011

10. Chiao C-C, Chubb C, Buresch KC, Barbosa A, Allen JJ, Mäthger LM, et al. Mottle camouflage patterns in cuttlefish: quantitative characterization and visual background stimuli that evoke them. J Exp Biol. 2010;213: 187 LP – 199. doi:10.1242/jeb.030247

11. Stoddard MC, Stevens M. Pattern mimicry of host eggs by the common cuckoo, as seen through a bird’s eye. Proc Biol Sci. 2010;277: 1387–1393. doi:10.1098/rspb.2009.2018

12. van den Berg CP, Troscianko J, Endler JA, Marshall NJ, Cheney KL. Quantitative Colour Pattern Analysis (QCPA): A comprehensive framework for the analysis of colour patterns in nature. Methods Ecol Evol. 2020;11: 316–332. doi:10.1111/2041-210X.13328

13. Troscianko J, Stevens M. Image calibration and analysis toolbox – a free software suite for objectively measuring reflectance, colour and pattern. Methods Ecol Evol. 2015;6: 1320–1331. doi:10.1111/2041-210X.12439

14. Stoddard MC, Kilner RM, Town C. Pattern recognition algorithm reveals how birds evolve individual egg pattern signatures. Nat Commun. 2014;5: 4117. doi:10.1038/ncomms5117

15. Spottiswoode CN, Stevens M. How to evade a coevolving brood parasite: egg discrimination versus egg variability as host defences. Proc Biol Sci. 2011;278: 3566–3573. doi:10.1098/rspb.2011.0401

16. Cassey P, Ewen JG, Blackburn TM, Hauber ME, Vorobyev M, Marshall NJ. Eggshell colour does not predict measures of maternal investment in eggs of *Turdus* thrushes. Naturwissenschaften. 2008;95: 713–721. doi:10.1007/s00114-008-0376-x

17. Green SD, Duarte RC, Kellett E, Alagaratnam N, Stevens M. Colour change and behavioural choice facilitate chameleon prawn camouflage against different seaweed backgrounds. Commun Biol. 2019;2: 230. doi:10.1038/s42003-019-0465-8

18. Hawkes MF, Duffy E, Joag R, Skeats A, Radwan J, Wedell N, et al. Sexual selection drives the evolution of male wing interference patterns. Proc Biol Sci. 2019;286: 20182850. doi:10.1098/rspb.2018.2850

19. Stevens M. Avian vision and egg colouration: concepts and measurements. Avian Biol Res. 2011;4: 168–184. doi:10.3184/175815511X13207790177958

20. Spottiswoode CN, Stevens M. Host-parasite arms races and rapid changes in bird egg appearance. Am Nat. 2012;179: 633–648. doi:10.1086/665031

21. Spottiswoode CN, Tong W, Jamie GA, Stryjewski KF, DaCosta JM, Kuras ER, et al. Genetic architecture facilitates then constrains adaptation in a host-parasite coevolutionary arms race. PNAS. 2022;119: e2121752119.

22. Reeve HK. The evolution of conspecific acceptance thresholds. Am Nat. 1989;133: 407–435. doi:10.1086/284926

23. Davies NB. Cuckoos, Cowbirds and Other Cheats. London: T. & A.D. Poyser; 2000.

24. Davies NB, Brooke MDL, Kacelnik A. Recognition errors and probability of parasitism determine whether reed warblers should accept or reject mimetic cuckoo eggs. Proc Biol Sci. 1996;263: 925–931. doi:10.1098/rspb.1996.0137

25. Caves EM, Dixit T, Colebrook-Robjent JFR, Hamusikili L, Stevens M, Thorogood R, et al. Hosts elevate either within-clutch consistency or between-clutch distinctiveness of egg phenotypes in defence against brood parasites. Proc Biol Sci. 2021;288: 20210326. doi:10.1098/rspb.2021.0326

26. Dixit T, Lund J, Fulford AJC, Apostol AL, Chen K-C, Tong W, et al. Chase-away evolution maintains imperfect mimicry in a brood parasite–host system despite rapid evolution of mimics. Nat Ecol Evol. 2023;7: 1978–1982. doi:10.1038/s41559-023-02232-4

27. Ryan P, Dean R. Croaking Cisticola (*Cisticola natalensis*), version 1.0. In: del Hoyo J, Elliott A, Christie DA, de Juana E, editors. Birds of the World. Ithaca, NY, USA: Cornell Lab of Ornithology; 2020. doi:https://doi.org/10.2173/bow.crocis1.01

28. Hanley D, Hauber ME, Holford M, Moya C, Spottiswoode CN, Dixit T. Pigment concentrations only partially predict avian eggshell colour mimicry in a polymorphic host–brood parasite system. Biol Lett. 2025;21: 20250112. doi:10.1098/rsbl.2025.0112
