# Supplementary material for: Categorical identity signatures can reduce host error rates during brood parasitism
Source: PLoS Biol. 2026 Feb 20;24(2):e3003667. doi: 10.1371/journal.pbio.3003667 (PMC12952647; doi:10.1371/journal.pbio.3003667)
Supplement: Attachment — Submitted filename: Response to reviewers.docx [file pbio.3003667.s004.docx]

Based on the reviews, we are likely to accept this manuscript for publication, provided you satisfactorily address the remaining points raised by the reviewers and the academic editor (copied after the reviewer reports). Please also make sure to address the following data and other policy-related requests.

Many thanks for the email and comments on the manuscript; we are delighted that our study received positive feedback. Below we respond to the editorial points and reviewers’/editor’s suggestions and comments.

IMPORTANT: please ensure that your next revision implements the following editorial points:

------------------

**Title:

To emphasize the main findings of your study, we suggest an alternative title for your paper. Is the following acceptable to you?

"Categorical identity signatures can reduce host error rates during brood parasitism"
 Thank you for this suggestion. We are happy to change the title as you suggest.

**Financial disclosure statement:

-- Please include in your statement the answer to the following question:

"Did the sponsors or funders play any role in the study design, data collection and analysis, decision to publish, or preparation of the manuscript?"

We have done this.
 
**Ethics: 

-- Please provide an approval number for AWERB, if applicable. Please also place the ethics information in a new, independent, and first sub-section of the Materials and Methods section called "Ethics statement".

Approval numbers are not applicable, since the applications were approved without providing an approval number. We have placed the ethics information in a new subsection as requested.

**Data:

-- Thank you for uploading your data and code to the Cambridge data repository. Please now make them available so that we can assess whether we need any additional information, prior to formal acceptance of your paper.

We asked the Cambridge data repository to make the data available; however, they say that “as your dataset is already ‘live’ (but under embargo) it is best if someone from the journal uses the Request a Copy option.”. This option would involve following the instructions here <https://www.openresearch.cam.ac.uk/cambridge/apollo/request-a-copy> with the dataset (<https://doi.org/10.17863/CAM.116928.2>). Apologies for this inconvenience – it just seems something required by the repository service. Would it be possible to use the Request a copy service to access the dataset?

-- Please note that we require that all of the numerical values underlying the plots in the main and supplementary figures are provided either in supplementary excel files or in the online repository.
NOTE: the numerical data provided should include all replicates AND the way in which the plotted mean and errors were derived (it should not present only the mean/average values).

All the numerical values underlying figures are provided in data sheets in the repository. The numerical data do indeed include all replicates from which any means, standard errors etc are derived.

 
-- Please cite the location of the data clearly in all relevant main and supplementary Figure legends, e.g. “The data underlying this Figure can be found in S1 Data” or “The data underlying this Figure can be found in <https://eur03.safelinks.protection.outlook.com/?url=https%3A%2F%2Fdoi.org%2F10.5281%2Fzenodo.XXXXX%25E2%2580%259D&data=05%7C02%7Ctd349%40universityofcambridgecloud.onmicrosoft.com%7Cb0a5561ada4043d737dc08de59296c09%7C49a50445bdfa4b79ade3547b4f3986e9%7C1%7C0%7C639046231621997676%7CUnknown%7CTWFpbGZsb3d8eyJFbXB0eU1hcGkiOnRydWUsIlYiOiIwLjAuMDAwMCIsIlAiOiJXaW4zMiIsIkFOIjoiTWFpbCIsIldUIjoyfQ%3D%3D%7C0%7C%7C%7C&sdata=AsHGsDG4siMLTCSxCm0CBiXSUKJr6WmLnz9z%2B1ozFgs%3D&reserved=0>

We have done this.

**Supplement:

We see that you have included supplemental methods, results, tables, and figures in your supporting information document. Because documents of this kind are not proofread and are rarely examined by readers, our preference is that these items are integrated into the main paper as much as possible.

Many thanks; we have revisited our choice of material to be placed in the supplemental materials. We believe that we have appropriately placed more tangential information, or information that would be required only by specialist readers interested in particular aspects of the methodology or results, in the SI. We think this strikes a good balance between making the main text clear and comprehensive, while avoiding over-filling it with information that supports the main conclusions but would nevertheless distract readers from the main messages of the paper. Of course, please let us know if you would like any particular item(s) moved to the main text, however.

-- Please upload the supplementary figures separately as supporting information files. Please include all supplementary item titles and legends at the end of the main text file, as well as supplementary tables.

We have done this.

-- As references that are only found in the supplement will not be included in citation records, please ensure that all references in the supplement are also included in the main references list.

We have done this.

 -- Supplementary files (e.g., excel). Please ensure that all data files are uploaded as 'Supporting Information' and are invariably referred to (in the manuscript, figure legends, and the Description field when uploading your files) using the following format verbatim: S1 Data, S2 Data, etc. Multiple panels of a single or even several figures can be included as multiple sheets in one excel file that is saved using exactly the following convention: S1_Data.xlsx (using an underscore).

We have uploaded our data files to a repository so do not include them as supplementary files. Thus, we have a supporting information document (with supplementary methods, results, and tables etc.) and supplementary figures that we have uploaded separately (as well as embedding them in the supporting information document).

 **Code availability:

-- Per journal policy, if you have generated any custom code during the course of this investigation, we require that you make it available without restrictions. Please ensure that the code is sufficiently well documented and reusable, and that your Data Statement in the Editorial Manager submission system accurately describes where your code can be found.
 
-- Please note that we cannot accept sole deposition of code in GitHub, as this could be changed after publication. However, you can archive this version of your publicly available GitHub code to Zenodo. Once you do this, it will generate a DOI number, which you will need to provide in the Data Accessibility Statement (you are welcome to also provide the GitHub access information). See the process for doing this here: <https://eur03.safelinks.protection.outlook.com/?url=https%3A%2F%2Fdocs.github.com%2Fen%2Frepositories%2Farchiving-a-github-repository%2Freferencing-and-citing-content&data=05%7C02%7Ctd349%40universityofcambridgecloud.onmicrosoft.com%7Cb0a5561ada4043d737dc08de59296c09%7C49a50445bdfa4b79ade3547b4f3986e9%7C1%7C0%7C639046231622026665%7CUnknown%7CTWFpbGZsb3d8eyJFbXB0eU1hcGkiOnRydWUsIlYiOiIwLjAuMDAwMCIsIlAiOiJXaW4zMiIsIkFOIjoiTWFpbCIsIldUIjoyfQ%3D%3D%7C0%7C%7C%7C&sdata=QBA7clJzhRadhPGZc26hCamMl8wrfi3FUlVoclfymkg%3D&reserved=0>

We have included appropriately documented code in the data repository (i.e. not in GitHub).
-----------------


As you address these items, please take this last chance to review your reference list to ensure that it is complete and correct. If you have cited papers that have been retracted, please include the rationale for doing so in the manuscript text, or remove these references and replace them with relevant current references. Any changes to the reference list should be mentioned in the cover letter that accompanies your revised manuscript.

Many thanks; we have done so.

Reviewer remarks:


Reviewer #1: This manuscript presents important new insights into the coevolutionary arms race between brood parasitic birds and their host species, combining empirical field studies with individual-based simulations. A primary conclusion of the paper is that there is not necessarily an inherent trade-off between optimizing Type 1 and Type 2 errors (that is, minimizing the rejection of the host's own eggs while at the same time maximizing the rejection of the parasite's eggs) as is usually predicted by signal detection theory. This novel conclusion is not only significant for understanding the coevolution of brood parasites and their hosts, but also more broadly applicable to other systems in which there is a potential trade-off between recognition of self and non-self. I expect this may become a widely-cited paper.

The empirical data and models presented here strongly support the manuscript's main conclusions. The manuscript is exceptionally well written. Truth be told, I do not have any major concerns with methods, analyses, or the interpretation of results. The following are some relatively minor suggestions for improving an already strong paper (in order as they occur in the manuscript):

Many thanks for your excellent summary of the paper and for your kind comments above – we are delighted that you enjoyed the paper. Many thanks also for your constructive and very helpful feedback below.

l. 79 - The term "multimodal" is appropriate to use to describe the distribution of potentially categorical traits. However, this term is now also commonly used in the context of signaling and animal communication to refer to the involvement of more than one sensory modality in a single functional signal (e.g., a bird simultaneously vocalizing while performing a visual display). So I'm (slightly) concerned that the sentence "Such traits are 'multimodel': their distribution included more than one modal value" might be initially misleading to some readers. Perhaps this (slight) concern of mine would be addressed by rewriting this sentence as "Such traits are 'multimodal,' meaning that the distribution of trait values has more than one peak."

This is a great point and one we were conscious of when using the term, hence us providing a definition. However, we agree that your rephrasing makes it clearer that this is a definition, and also makes the definition easier to understand, and have incorporated it as you suggest.

l. 110 - Given that the previous two sentences are referring to hypotheticals, I think it would be good to explicitly state that the negative correlation between consistency and distinctiveness in eggs is an empirical observation. Something like "However, empirical observations have shown that consistency and distinctiveness in egg appearance…"

Again, thank you for this very helpful point. We have edited the sentence as you suggest.

Fig 1 - This is a very useful figure! But I found myself wanting to see a similar array of eggs for the croaking cisticola, given that its eggs are neither apparently categorical (as illustrated for the zitting cisticola in panel a) nor apparently continuous (as illustrated for the tawny-flanked prinia in panel b). Perhaps the combination of categorical and continuous traits would be less visually obvious (or harder to pull out from looking at the images, but wouldn't that help make the point?

This is a very good thought and we agree! However (and apologies for this), the reason for not including a similar photo of croaking cisticola eggs (and red-faced cisticola eggs) is that taking such images requires simultaneously having available a large number of eggs from different host nests (which we collected as part of experiments) to illustrate the variation present. For the slightly less common species, red-faced and croaking cisticolas, we did not have enough collected eggs at the same time to take such a photo. Thus, although ideally we would include a ‘circle’ illustrating species-wide variation for each host, we hope that Figure 1 illustrates the extremes (fully categorical; fully continuous) while Figure 2 provides the exact distributions for all four species, along with illustrative images of individual eggs covering the range of phenotypes observed in these hosts.

l. 164 - I realize that PAM cluster analysis is explained in the Methods, but given that this isn't a widely used method, I think it would be good to spell the name out when first mentioned here in the text since the acronym alone seems like jargon.

We fully agree – apologies for this oversight. We have added the full term when first used as you suggest.

ll. 414-426 - Two point about this paragraph: First, after reading this several times I wasn't completely sure if the authors are suggesting that categorical variation of traits is the ancestral state with continuous variation inherently being the derived state. If that's the case, it would be good to state such more explicitly. Second, if categorical variation is ancestral, I wonder if it isn't equally important to ask why zitting cistolas have maintained categorical variation in the face of the selective factors that lead to continuous variation as described in this paragraph. I certainly may be misunderstanding something here, but this was the one point in the paper where I didn't fully follow the argument being made, so perhaps this could be clarified.

Many thanks for this comment and for pointing out that it was somewhat unclear. The point here is less about ancestral states and more about why categorical variation has either (a) not evolved or (b) been lost in the hosts that exhibit continuous variation. We have edited the beginning of the paragraph to make this explicit, which we believe clarifies your excellent question. The text now reads: “Some hosts, such as prinias and red-faced cisticolas, exhibit continuous variation. This indicates that either categorical variation never evolved in these species, or that it evolved but was then lost, which is perhaps surprising given that categorical variation leads to lower frequencies of Type I and Type II errors. One reason that categorical variation may not inevitably evolve is that trait distributions are properties of populations, not heritable properties of individuals, so cannot directly respond to selection.”

For your second point, we make no assumptions about ancestral states (though one might imagine categorical variation to be the first step in intra-specific diversification in hosts, since it likely has a simpler genetic basis and requires fewer novel mutations to spread than continuous variation). We agree that it might be important to ask why zitting cisticolas have maintained categorical variation, but were more concerned with why (seemingly highly adaptive) categorical variation is not universal. To briefly address your question, though, we now add that one explanation for why zitting cisticolas exhibit categorical variation is that it “has not *yet* been lost”, even if it eventually will be.

l. 581 -I understand that the terms "gen" or "gente" are used to refer to host-adapted lineages in the brood parasitism literature. I don't think the term is widely used in other areas of biology (although it is more so in anthropology), so it stands out here a some field-specific jargon. Maybe this should be described more completely here for the non-specialist. (This also appears in one or two places in the supplementary materials.)

Many thanks for this point – we have now explained the term ‘gentes’ in the sentence you refer to: “…differing degrees of mimetic accuracy and phenotype distributions by the lineages (known as ‘gentes’) that specialise on each host”, and retained the brief explanation in the introduction. Similarly, we explain it where it first appears in the supplementary materials.

Reviewer #2: This is an exciting manuscript that shows how far the field has come since the first work to codify egg rejection, thresholds, and error rates by Davies et al. 1996 (Proc B). Sophisticated measures of egg coloration and patterning are combined with statistical approaches to demonstrate continuous versus clustered categorical 'signatures' in several host species of the brood parasitic African cuckoo finch. By embedding these measures within the results of field experiments, these data therefore not only confirm our human-vision 'hunches' of egg phenotypes, but also the properties that matter to the hosts themselves. The authors then go on to use these rejection decisions by hosts to explore error rates - accepting a foreign egg (Type II) vs. rejecting their own egg (Type I) and identify that the error rates differ among host species depending on which signature strategy has been adopted. Finally, simulations are used to identify the signal distances at which rejection errors occur, depending on whether the receiver uses a threshold or category-based decision AND what the underlying variation in the signal has evolved to be.

The manuscript is elegantly written and for the most part (see specific comments below), much of the methodological decisions and theoretical basis for the work is explained very clearly. The most significant finding is that category-based perception is most effective when trait variation can also be categorised - this raises an interesting question as to which evolves first, perceptual ability or traits. Coevolution with a mimicking antagonist complicates this, however, as there may also be selection for intermediate traits to avoid the mimic. These are questions and problems that are generalisable across coevolving systems - and which are highlighted and nicely discussed in the manuscript.

Many thanks for your excellent summary of the manuscript and for the kind comments about it (and the further questions it leads to). Thank you also for your suggestions below, which we have found very helpful in revising the MS.

Specific comments:
abstract sentence 3: has redundancy  - if categories are not discrete, would this differ conceptually from continuous variation for recognition? Suggest for sake of clarity to modify this and the following sentence to read 'Here we test how discrete categorical distributions may influence fundamental trade-offs in signal recognition, using a brood-parasite host system.'

That you for this comment. There is not actually redundancy here because perception of signals as being categorical is not the same thing as the variation being categorical (see Fig 4, where we simulate categorical distributions being perceived in a both non-category-based and category-based manners). However, we agree that the phrasing as it currently is does make the sentence sound redundant, especially as readers of the abstract will likely lack the context necessary for understanding the distinction. We have therefore taken your suggestion for this sentence.

Line 59: consider adding emphasis to the and (e.g. by italics) to make it clearer that this is the surprising aspect of the results

This is a great idea, thank you! We have done so – assuming that this is acceptable according to journal formatting.

Lines 76 - 80: here the use of gametes as an example to explain categorically distributed traits is useful, but I wonder if this is perhaps a little too obvious (although I understand the motivation to help relate the study to a broader audience). Here it may be more useful and pertinent to the study question to consider using a well-known example from one of the contexts mentioned for trait perception - this would help the reader make the conceptual link between these main concepts.

Thank you for this suggestion. We agree that the gamete example is a little obvious – that is partly why we used it, to ensure the explanation is clear for a broad audience – but, given your suggestion, we have changed this to another quite general and equally understandable example: “colour polymorphisms where each individual belongs to a particular colour ‘morph’”.

Lines 80-82: given the space given to describe a categorical trait, it is surprising that categorical perception is not as clearly explained. Three examples are referred to, but readers would still need to go to those citations to figure out what is meant by categorical trait perception.

Thank you for this comment. In response, we now provide a brief definition: “(where animals respond to stimuli as though they belong in discrete categories [4])”. Categories have been defined previously, so we think that this definition should suffice.

Lines 167-170: this wording makes it seem like the categories being consistent with our perception is a key result - is this intended? It makes it easier for future studies to refer back to perhaps, but I don't think that this deserves being repeated verbatim for each species. Perhaps it could be reworded such that the variation clusters and can therefore be described categorically (or not) (Figure 2), and then a comment that the results match human perception could be included in the otherwise very short 'paragraph' about trait correlations (lines 220-221) and avoid it becoming lost.

We like this suggestion very much and have implemented it as you suggest: for each species, we state the key result (e.g. “Thus, red-faced cisticola eggs showed continuous variation in traits predicting rejection”) and specify the congruence with human perception of their phenotypes in the paragraph about trait correlations. Thank you!

Figure 3: 'dumbbell plots' are not particularly informative as to the distribution of the data. Consider using e.g. violin plots for the experimental data so that the data is more clearly visualised.

Many thanks for this suggestion. We do not use violin plots or boxplots here because the experimental data are binary (either an error was made or it wasn’t), and the figure shows proportions of errors made. Thus, the distribution of the data would not be informative, as is typically the case for data relating to the frequencies of binary outcomes (see e.g. Figure 2 of Cuthill et al 2016 PNAS). Therefore, we propose to retain the figure as a bar plot.

Line 287: the assumption that Type I errors are only possible if a parasitic egg has been rejected is a little puzzling as there is some empirical evidence from other host species showing that they may remove their own eggs accidentally, leaving a parasitic egg behind (although it is possible that this could be an artefact of the use of artificial egg models). Could this assumption be clarified?

We apologise for the confusion here: the point is that Type I errors can indeed occur in unparasitised nests, but can only have an effect in *parasitised* nests if the parasite egg has been rejected (otherwise the whole clutch is doomed regardless of whether the host rejects any of its eggs). We now clarify this by stating in Line 292 onwards: “In these simulations, Type I errors (rejecting own eggs) are only considered to have an effect in either unparasitised nests or in parasitised nests when a parasitic egg has already been rejected. This is because if a parasitic egg is accepted (i.e. if a Type II error is made), then any Type I errors have no effect since the whole host clutch is in any case doomed (see Methods and SI §h).”.

Simulations, text and Figure 4: I found this section a little more challenging to follow, requiring several reads to figure out what was done and why. Perhaps consider explaining explicitly in the figure legend what 'threshold distance' refers to as this is likely to trip up more naive readers, and because it also shares 'threshold' with the rejection mechanism. I got distracted by the use of the same colours across the rows of this figure, at first thinking that it didn't make sense to simulate the variation for categorical signals to have so little variation. Perhaps (d)-(f) could use different shades of red and blue to highlight that these reflect different rejection mechanisms of the respective types of signature variation.

Thank you for this comment – we recognise that the simulation component is necessarily complex, and appreciate the suggestions for making it clearer. In particular, we have implemented your suggestion regarding the figure legend, in order that it should stand on its own for readers who wish to just understand the key result rather than focussing on the details of the simulations. Specifically, we state that “In panels (b), (c), (e), and (f), ‘threshold distance’ refers to the distance whereby eggs differing from the host phenotype by more than that distance are rejected.”

Thank you also for your comment about the colours. We agree that there is a distinction between (a)-(c) and (d)-(f). However, the simulations are implemented identically, except the specific difference that in (d)-(f), both intra-clutch variation and variation around each peak are set to 0 such that category-based rejection can be modelled (a point that we emphasise in the both the figure legend and the title above each row). Threshold distances and rejection are implemented identically, as are all other parameters. In both rows, the continuous populations are modelled identically (as we now state explicitly in Line 337-8: “a continuous population (modelled in an identical manner to the continuous population described in (i); see Figure 4a and 4c) in (i)”). Therefore, we believe that changing the colours would add more confusion, as the meaning of the colour code (which is used to distinguish the two populations) has not changed.

In response to your more general point about finding this section difficult to understand, we have also made several minor edits to phrasing with an aim to further clarifying it. For instance, we add extra context explaining the fundamental take-home message and rationale of this section in the second sentence of the section (Line 280 onwards): “Effectively, the simulations calculate expected error rates for different host trait distributions, and illustrate two mechanisms by which categorical distributions might result in hosts making fewer Type I and Type II errors.”

Additional comments from the Academic Editor [lightly edited]:

I have a few very minor comments for the authors

Line 101 'depend upon'
Table 1 - add sample sizes
Line 308 change 'mass' to 'number'

Thank you for these three suggestions; we have implemented them as requested.

Line 342. I understand the maths, but there is something strange about referring to the overall Type II error rate and including nests (the proportion being 1-p) where there was no opportunity for such an error because there were no parasitic eggs. A simple solution is to refer to the NUMBER of Type II errors (i.e. drop the 'rate'). Or just be clear that this is not the actual rate of errors when confronted by a parasitic egg.

Thank you for this point, and we agree that it is arguably strange to incorporate unparasitized nests in a Type II error rate estimate. The reason we do this here is because the success rate across a population necessarily depends on the combination of how many Type II errors are made in parasitised nests and how many nests are parasitised. Excluding the latter would make a slightly more ‘normal’ definition of Type II error rate, but would ignore a crucial aspect of the impact of Type II errors on overall success.

Therefore, we propose to take your second suggestion and be clear that the actual rate of errors when confronted by a parasitic egg is 1/n, while P/n refers to the overall acceptance rate of parasitic eggs across the population. Specifically, we now state that: “Thus, the probability of a Type II error in a parasitised nest is 1/n. Across the whole host population, the overall frequency of Type II errors across all nests (both parasitised and unparasitised) is P/n, where P is the parasitism rate (Figure 4f). In Figure 4d–f, for example, P = 0.5 and n = 2, so the Type II error rate under category-based rejection is 0.25 (i.e. 25% of host nests will involve a Type II error; Figure 4f).”

Finally, I think the authors could protect themselves against a valid criticism by noting that this is a comparison of only a few species that focuses on egg trait distributions as the cause of differences among species in egg rejection/acceptance rates. It might be unlikely, but it is not inconceivable, that some other factor that differs among the species causes differences among these species in rejection/acceptance rates. To take a rather silly example, maybe all the species are equally good at discrimination between host and parasite eggs. But the species might vary in their propensity to act on this information because removing eggs increases the risk of total nest failure if predators detect removed eggs and use this information to locate nests. If the occurrence of such predators differed across species it could cause differences in type 1 and type 2 error rates. I am sure the authors can think of a more biological plausible alternate explanation for their findings.

Thank you for this comment. Indeed, this is a comparison of few species and (while it is difficult to think of a specific factor that would explain how zitting cisticolas produce low rates of *both* types of error) it may be that there is some other factor that could cause this. Your example is a very nice one, though in the present study system it seems unlikely, because all the hosts studied dispose of rejected eggs far from their nests. Nevertheless, we appreciate the general point here, and now state in the first paragraph that “Overall, this study, albeit necessarily focussing on only four host species, illustrates the role of trait distribution shapes in influencing ecologically- and evolutionarily-relevant behaviours.”

Moreover, we have now added a paragraph to the Discussion addressing the possibility of alternative explanations which reads: “Although our study provides evidence for association between categorical variation and low error rates, and mechanisms by which this should occur, such a comparative study cannot directly test for causality. Specifically, we cannot definitively rule out that some other species-specific factors could generate the low error rates seen in zitting cisticolas. However, species-specific differences in traits that affect error rates, such as recognition ability or the degree of inter- and intra-clutch variation, are unlikely to explain why zitting cisticolas exhibit low rates of *both* types of error. This is because varying these factors should concurrently influence the rates of both errors in opposite directions (as discussed in the introduction, with empirical evidence from the present system provided in SI §e; also see [22,25]). Further evidence for the role of categorical variation and category-based perception in reducing error rates might be gathered by empirically testing for these associations in other systems.”
